# Home-Based Frailty Prevention Program for Older Women Participants of Kayoi-No-Ba during the COVID-19 Pandemic: A Feasibility Study

**DOI:** 10.3390/ijerph19116609

**Published:** 2022-05-28

**Authors:** Ryota Watanabe, Masayo Kojima, Mikako Yasuoka, Chieko Kimura, Koto Kamiji, Takahiro Otani, Shoko Tsujimura, Hitomi Fujita, Akane Nogimura, Sae Ozeki, Aiko Osawa, Hidenori Arai

**Affiliations:** 1 Department of Frailty Research, Research Institute, National Center for Geriatrics and Gerontology, Obu 474-8511, Aichi, Japan; masayok@ncgg.go.jp (M.K.); yasuoka@ncgg.go.jp (M.Y.); kamiji.koto@shizuoka.ac.jp (K.K.); otani@med.nagoya-cu.ac.jp (T.O.); t-shoko@sozo.ac.jp (S.T.); hitomifu@n-fukushi.ac.jp (H.F.); nogi.nene@gmail.com (A.N.); ozekisae146@gmail.com (S.O.); 2 Department of Social Preventive Medical Sciences, Center for Preventive Medical Sciences, Chiba University, Chiba 263-8522, Chiba, Japan; 3 Senior Care Division, Welfare Department, Handa City, Handa 475-8666, Aichi, Japan; kimura.c.22@gmail.com; 4 Center for Professional Development of Teachers, Shizuoka University, Shizuoka 422-8529, Shizuoka, Japan; 5 Department of Public Health, Nagoya City University Graduate School of Medical Sciences, Nagoya 467-8601, Aichi, Japan; 6 Department of Health Sciences, Toyohashi Sozo University, Toyohashi 440-8511, Aichi, Japan; 7 Department of Rehabilitation, Faculty of Health Sciences, Nihon Fukushi University, Handa 475-0012, Aichi, Japan; 8 Division of Psychiatry and Cognitive-Behavioral Medicine, Nagoya City University Graduate School of Medical Sciences, Nagoya 467-8601, Aichi, Japan; 9 Department of Rehabilitation Medicine, Hospital, National Center for Geriatrics and Gerontology, Obu 474-8511, Aichi, Japan; aiko@ncgg.go.jp; 10 National Center for Geriatrics and Gerontology, Obu 474-8511, Aichi, Japan; harai@ncgg.go.jp

**Keywords:** resistance training, five times sit-to-stand test, National Center for Geriatrics and Gerontology Home Exercise Program for Older People, home-based exercise

## Abstract

This study presents a single-arm intervention that aimed to determine the feasibility of a three-month home-based exercise program to prevent the progression of frailty during COVID-19. We recruited four groups of Kayoi-no-ba, or community salons for frailty prevention, and a total of 69 community-dwelling older women who belonged to one of the Kayoi-no-ba in a preliminary study for a follow-up study. The intervention program was developed on the basis of the 5A approach, and the focus group by the volunteer leaders of Kayoi-no-ba. We adapted the National Center for Geriatrics and Gerontology Home Exercise Program for Older People for 10-min daily home-based exercise. For feasibility outcomes, 91.3% of the participants completed the intervention program, whereas the percentage of exercise performed was 86.5% during the intervention period. For health-related outcomes, the five times sit-to-stand test exhibited significant improvement after the intervention. The results of feasibility outcomes indicate that the program may be feasible due to the high rates of completion and exercise performed. Additionally, improvement was noted for the health indicators of the five times sit-to-stand test, which may help prevent frailty. The feasibility trial has provided the necessary data to design a future-cluster randomized controlled trial.

## 1. Introduction

Kayoi-no-ba has been attracting attention as a means of preventing the onset and progression of frailty among community-dwelling older adults in Japan [1]. The term Kayoi-no-ba broadly refers to activities focused on resident-centered frailty prevention through physical exercise, hobbies, or other activities [2]. Moreover, it refers to places where older adults can interact with their neighbors regularly. In addition, Kayoi-no-ba is operated mainly by volunteer leaders with the financial support of the local government. However, the COVID-19 pandemic [3], which broke out in Wuhan, China in December 2019, forced many Kayoi-no-ba to be closed [4]. Previous studies have disclosed that community-dwelling older adults gained less opportunities to interact with others and less physical activity [5], such that concerns have emerged about the increased risk of frailty [6]. Alternatively, scholars have demonstrated in a randomized control trial (RCT) that a home-based exercise program implemented during the isolation imposed by COVID-19 led to the improvement of muscle strength in the lower limb [7]. Thus, the need exists to promote exercise programs that older adults can perform at home.

In May 2020, the National Center for Geriatrics and Gerontology published an information booklet entitled “National Center for Geriatrics and Gerontology Home Exercise Program for Older People (NCGG-HEPOP) 2020” to promote the prevention of frailty affected by restrictions on outings due to COVID-19. The booklet describes precautions in daily life and exercise methods, including resistance training and stretching exercises, which can be performed at home with the objective of maintaining mental and physical functions during the COVID-19 pandemic [8,9]. A meta-analysis of 25 intervention trials revealed that resistance training improved physical function, including grip strength, lower limb strength, and walking speed, among pre-frail, frail, and sarcopenic individuals [10]. In addition, a systematic review of intervention studies demonstrated that the effectiveness of exercise programs for pre-frail and frail individuals revealed high levels of improvement in frailty when conducted in groups [11].

Therefore, the researchers are convinced that providing intervention for the group of Kayoi-no-ba to maintain connection with peers for frailty prevention is important. We developed a frailty prevention program adapting the NCGG-HEPOP for the participants of Kayoi-no-ba. As the framework of the intervention program, we adopted the 5A approach, which is composed of five stages; i.e., Ask, Advise, Agree, Assist, and Arrange, and partially originates from the 4A (ask, advise, assist, and arrange) developed by the National Cancer Institute for smoking cessation treatment [12]. It is currently being applied to promote behavioral change and improve lifestyle habits in patients with chronic diseases such as obesity and diabetes [13]. To explore the possible barriers and promoters of the intervention, a focus group was conducted inviting nine volunteer leaders who manage the Kayoi-no-ba, as well as two public health nurses. According to the thematic analysis of the focus group under the framework of the 5A approach, the study extracted the following elements as the core of the intervention program: Ask: assessment of the current status; Advise and Agree: setting goals and approvement from leaders; Assist: recording exercise performance and sharing it within the group; and Arrange: follow-up from leaders by weekly phone call and support from specialists [14].

This study aims to determine the feasibility of a frailty prevention program based on Kayoi-no-ba. We are planning a cluster RCT study to verify the effectiveness of the program after this preliminary study.

## 2. Materials and Methods

### 2.1. Study Design

This is a feasibility study with one arm intervention. Feasibility studies have the ability to test the methodology of intervention studies that are planned to be conducted subsequently [15]. We planned this preliminary study to examine the feasibility of the program which we had newly developed, and to collect basic data and identify the barriers to conducting a cluster RCT. The study protocol was registered under UMIN-CTR (R000049753).

### 2.2. Participants and Setting

This study was conducted at the Kayoi-no-ba in Handa City, Aichi, Japan, where seniors in the community can gather and interact. Kayoi-no-ba meetings are held two to four times per month for the disability prevention program, which consists of exercise, brain training, and recreation. Leaders in each Kayoi-no-ba manage and facilitate the program content. The inclusion criteria were older adults who are registered and enrolled in the Kayoi-no-ba invited by the public health nurses. In September 2020, public health nurses invited the leaders of four Kayoi-no-ba to participate in a preliminary study. A total of 78 community-dwelling older adults belonging to one of the four Kayoi-no-ba were recruited for the current intervention. The exclusion criteria were (1) those without research consent, (2) those with declined cognitive function and considered unable to complete the program, (3) those with high risk of falling due to a decline in physical function, and (4) men. The reason why men were excluded is that there were only a few men among the Kayoi-no-ba participants, and the results could not be fully significant.

### 2.3. Intervention

The participants were instructed to perform 10 min of a home-based exercise program every day for three months. Table 1 presents a summary of the intervention program. First, the participants are tasked to read the NCGG-HEPOP booklets, which describe exercise methods distributed by the leader, and select an exercise program based on the flowcharts. This flowchart was established with reference to the questionnaires that have been reported to have validity in predicting the incidence of disability and death [8,16,17,18,19]. The results of this flowchart determined the most suitable exercise programs [2,8]. The original HEPOP includes a cogni-pack and a nutrition improvement pack, which the study did not use, as we focused on the physical exercise program [14].

In the next step, the leader distributed a printed sheet describing the selected exercise program [20] to each participant. Each exercise menu contained stretching and resistance training and can be completed in 10 min [20]. Details of the exercises are shown in Table 2. An intensity of 8–12 Repetition Maximum is recommended for resistance exercise to increase muscular strength, mass, and endurance [21]. Meanwhile, beginners performing high intensity training can cause pain [22] and decrease exercise adherence [23]. In addition, it is known that muscle strengthening can be obtained even with low intensity exercises [24], so the amount of load used in NCGG-HEPOP is based on an intensity that a typical older person would perceive as around 4 on the modified Borg scale [25]. In addition, a QR code was attached to the form so that the video could be viewed from there. The participants were expected to perform the 10-min daily exercise at home according to the exercise menu and were requested to write down their goals of the month on the form and mark the calendar if they conducted the exercise per day. The participants were asked to submit the exercise record sheet to the leader on the day of the Kayoi-no-ba meeting. Moreover, the leaders are expected to encourage the participants to continue the exercise and check the exercise record sheet once per week. The leader contacted the participants via phone or e-mail if they were absent from the Kayoi-no-ba meeting or on a week when no Kayoi-no-ba meeting was held. In addition, the research office answered questions from leaders as required and provided support.

### 2.4. Outcomes

#### 2.4.1. Feasibility Outcomes

The feasibility outcomes were retention rate, percentage of leaders and participants undergoing intervention, satisfaction of the participants, facilitation, and barriers to intervention. These data were collected during and at the end of the intervention.

Retention rate was defined as the percentage of the number of participants who continued until the end of the intervention from the number of enrolled participants. The percentage of leaders that implemented the intervention was calculated as the number of the recorded sheets of the participants confirmed by the leaders out of the expected number during the intervention period. The percentage of participants that underwent the intervention was calculated as the dates when the participants performed the exercise for the entire duration of the intervention period confirmed by the recorded sheets. The level of satisfaction of the participants was assessed using a five-point Likert-type scale at the end of the intervention. In addition, volunteer leaders were also interviewed after the intervention to determine the factors that facilitate and barriers to participation in the intervention.

#### 2.4.2. Effectiveness Outcomes

Effectiveness outcomes were changes in the prevalence of frailty, health-related quality of life, changes in self-reported frequency of exercise, physical function, and physical activity. These data were collected before, during, and after the intervention.

The prevalence of frailty was determined using the score of the well-validated self-reported questionnaire called the Kihon Checklist, which is a 25-item with two Yes/No options, a Comprehensive Geriatric Assessment that evaluates the functions of older people [16]; scores of 0–3, 4–7, and 8 were designated as robust, pre-frail, and frail, respectively [26]. This indicator has been associated with the criteria of the Cardiovascular Health Study [27]; the sensitivity and specificity for frailty were reported to be 89.5% and 78.3%, respectively, and for pre-frailty were 70.3% and 80.7%, respectively [26]. In addition, its predictive validity for the incidence of disability and deaths in community-dwelling older adults three years later has also been confirmed [18]. The index of the risk assessment scale was calculated by extracting ten important indicators from the Kihon Checklist and assigning a score to each indicator to obtain the total score. This set of indicators has been validated in terms of predicting the incidence of disability within three years for community-dwelling older people [28].

For the assessment of the health-related quality of life, we used the EuroQol-5D-5L (EQ-5D-5L). The index consists of five dimensions (i.e., mobility, self-care, usual activities, pain/discomfort, and anxiety/depression), where each dimension is rated as no problems, slight problems, moderate problems, severe problems, and extreme problems. The values in this study were calculated using the conversion formula for the Japanese population [29], where death takes a value of 0, whereas perfect health takes a value of 1.

The change in exercise frequency was confirmed at the end of the intervention using a questionnaire adapted and modified from the questionnaire of the Japan Sports Promotion Center for comparison among community-dwelling older adults [30].

To evaluate physical function, the study measured grip strength and the five times sit-to-stand test as indicators of muscle strength measurements before and after the intervention. Grip strength was measured using a Smedley grip strength meter (T.K.K. 5001, Takei Scientific Instruments). The width of the dynamometer was set at the participant’s second interphalangeal joint, with a stationary standing position with legs naturally open and arms hanging down beside the body [31]. Considering the burden on the subject, the number of measurements was one on each side. The test-retest reliability was maintained even with only one measurement [32]. The maximum value of grip strength was used [33]. The five times sit-to-stand test was measured by repeating five consecutive standing and sitting movements from a chair sitting position as quickly as possible. The study measured the time required from the start of the movement to the full standing position after the completion of the five standing movements [34]. A professional member of staff conducted both evaluations. For the five times sit-to-stand test, the measurement was conducted in two out of four Kayoi-no-ba.

Physical activity was adopted as the duration of activities from the moderate-vigorous physical activity (MVPA), which was 3.0 METs or more [35]. For the measurement, the study used a tri-axial accelerometer (Active style Pro HJA-750C, OMRON Healthcare) with an epoch length set to 60 s, taken before the intervention and at week 8 (±1 week) after the start of the intervention. During this period, the participants were instructed to wear the device from waking to bedtime across seven days. For inclusion in the analysis, participants wore the accelerometer for at least four days and for at least 10 h/day of valid wear time. The definition of non-wearing time is the total time that the activity intensity is below the detection threshold, whereas activity is considered to be 60 min or more of continuous zeros [36].

### 2.5. Sample Size

The main purpose of this study was to determine the feasibility of this program. For this reason, no sample size calculations were performed. The number of participants is within the range of a previous feasibility study [37].

### 2.6. Statistical Analysis

The feasibility outcomes were validated using descriptive statistics. For effectiveness outcomes, comparative tests were performed before and after the intervention. Based on the results of the Shapiro–Wilk test, a paired *t*-test and Wilcoxon rank-sum test were conducted, and for categorical variables the McNemar test was conducted. To investigate whether the health status of the participants affected the outcome, the participants were stratified using robust pre-frail/frail. All statistical analyses were performed using IBM SPSS Statistics 27.0 (IBM Corporation, Armonk, NY, USA), with *p* < 0.05 and *p* < 0.10 indicating the statistical significance and the statistical significance trend, respectively.

## 3. Results

We recruited four groups of Kayoi-no-ba with a total of 78 community-dwelling older adults attending the information sessions. Nine were excluded due to the lack of consent (*n* = 1), cognitive impairment (*n* = 4) and men (*n* = 4) which resulted in 69 participants. During the intervention period, six participants dropped out due to health problems, whereas 63 (91.3%) completed the program. No participant pointed to a difficulty in participating in this program due to the content of the intervention.

Table 3 presents the baseline characteristics of the participants. The mean age was 79.5 ± 5.3 years; 39.7% were robust, whereas 60.3% were pre-frail or frail.

Table 4 illustrates the results of the intervention. The percentage of participants that completed the intervention was 86.5 ± 20.0% and 74.6 ± 21.7% for the leaders on average. The percentage of satisfied participants was 58.7%. When comparing the robust and pre-frail/frail participants, the percentage of implementation was higher for the robust group.

Based on the interviews with the leaders, the study identified the facilitating and barrier factors of the intervention as follows. The use of the record sheet was a facilitating factor in maintaining the motivation of the participants to continue the exercise. In addition, the motivation of the participants could be encouraged by the improvement in physical function and by participating with their group members. As a barrier factor, several leaders complained that individually calling the participants to check their performance was a stress. However, they felt rewarded by becoming acquainted with aspects of the participants through phone calls. Additionally, some participants exercised using their personal methods, because they lacked access to the video through the QR code. Moreover, the leaders recognized that the participants performed the exercise much more positively than they had expected.

Table 5 presents a comparison between before and after the intervention of the health indicators and changes in exercise frequency. In summary, the five times sit-to-stand test indicated a significant improvement, whereas grip strength exhibited a significant trend toward improvement for robustness. In addition, a significant improvement was observed using the risk assessment scale among the pre-frailty/frailty groups. Alternatively, the EQ-5D-5L produced worse results for robustness. The amount of physical activity assessed by the accelerometer remained the same. Approximately half of the participants reported an increase in the frequency of exercise after the intervention.

## 4. Discussion

This study examined the feasibility of the three-month home-based exercise program in preventing the progression of frailty. We confirmed high retention rates and a high percentage for implementing the intervention, which indicate feasibility. Physical function evaluated for potential effectiveness also improved, whereas the frequency of exercise increased. For the next cluster RCT, the following points can be noted: adapting the exercise intensity for each participant, providing clear instructions for the exercise, promoting an exercise program for frail participants who are unable to maintain exercise, and lessening the burden of leaders in monitoring the participants once a week. These pointers were considered to be within the range of possible improvements.

A previous study that assessed community-dwelling older adults for frailty using the Kihon Checklist shows that 17.2% were frail [18], compared to 29.5% in the present study. In a study of the Kayoi-no-ba, the percentage of those aged 75 or older was reported to be 37.0–59.5% [38,39], and 82.5% participants in this study were older. The current study participants are older than in previous studies and have a higher prevalence of frailty.

A systematic review of intervention studies of home-based resistance training reported an average continuation rate of 85.0% and an average overall compliance (% completed workouts) rate of 69.5% [40], with a continuation rate of 91.3% and an exercise compliance rate of 86.5%, which are higher than those of the current study. Compared with the 100% satisfaction of the respondents in the health education study for the prevention of care for community-dwelling older adults [41], the percentage of satisfied respondents in this study (58.7%) was low. Alternatively, the number of dissatisfied respondents was extremely low (3.2%). Those who did not respond “satisfied” tended to be older, frail, and with low percentages of exercise performed. More detailed support is needed for those with higher age and frailty at the baseline assessment to help them achieve a satisfaction through the home-based program. In terms of the change in exercise frequency, a survey conducted by the Sports Agency in 2019 prior to the COVID-19 pandemic indicated that 15.3–16.4% of the respondents aged 60–70 years women reported an increase, whereas 17.3–17.8% reported a decrease when asked about the change in their exercise frequency compared to the previous year [30]. In the post-intervention survey of the current study, exercise frequency increased in 47.6% of the participants, and did not decrease, which suggests that the current intervention increased exercise frequency.

In the interviews after the intervention, the comments from the leaders were generally positive. A few felt that weekly phone calls were a burden, but found that strengthening the connection of the group is a worthy effort. Limiting phone calls only to the beginning of the program may reduce the stress of leaders and improve implementation in future dissemination, despite the currently high rate (74.6%).

The retention rate and percentage of exercise performed to evaluate the feasibility of the current study were better than those of previous research. A systematic review that assessed the factors associated with participation in resistance training has suggested four key points for promoting resistance training [23]; i.e., (1) targeting people with health problems, (2) providing enjoyment and helping to build self-efficacy, (3) obtaining support from others, and (4) planning and self-monitoring [23]. The current program contains these four key points, which enabled the achievement of high feasibility. However, contrary to our expectation, the percentage of the exercise performed was lower among the pre-frail/frail group than the robust participants. Thus, they may need extensive support to promote self-efficacy, with encouragement from leaders to participate.

The present study observed a significant improvement in the physical function assessed using the five times sit-to-stand test. This result is consistent with a meta-analysis that reported improvements in physical function, including grip strength and the five times sit-to-stand test by resistance training among pre-frail/frail individuals [10]. In addition, the risk assessment scale demonstrated an improvement among the pre-frail/frail participants. The scale is an early indicator of disability [28], and this intervention may be able to delay disability. The EQ5D5L unexpectedly tended to worsen among the robust sub-group. The mean value of Japanese women aged 70 years or older in the EQ5D5L was reported at 0.828 ± 0.202 [42], whereas the mean EQ5D5L for robustness in the current study was 0.936 ± 0.059 and 0.900 ± 0.062 before and after the intervention, respectively. Both values are higher than the average of the general population. Thus, regression to the mean [43] may be the reason underlying this result. As such, verifying the effect in the next cluster of RCT is necessary.

Although the percentage of the performed exercise was high, improvement in the physical activity quantity was not observed, unexpectedly. According to the interview results, the participants exercised using their personal style, because they were unable to watch the video. Therefore, a possibility exists that changes in the amount of physical activity assessed using the MVPA could not be captured, because the participants performed exercises with insufficient intensity. In the next cluster RCT, improving the method of the environmental setting of the video is necessary to improve the quality of the exercise method and to confirm the exercise method by experts.

The strength of this study is that we showed the feasibility of the home-based exercise program while maintaining a connection with peers in the midst of COVID-19. However, the current study also includes the following limitations. First, the study did not set a target value in advance of proceeding with the cluster RCT. However, the retention rate and percentage of the performed exercise, which formed the feasibility index, were considerably high. Therefore, the study infers that the current program was acceptable. Second, this study is a feasibility study with one arm intervention. Therefore, it is not sufficient to objectively evaluate effectiveness. We are planning the cluster RCT to determine effectiveness of this program. Third, there are issues regarding the generalizability of the results of this study. As the participants of this study are limited to women in a city, it is not possible to discuss the applicability of this study to other regions or men. However, more than 80% of those attending the Kayoi-no-ba are women [38,39], which could be applied to many Kayoi-no-ba. In addition, the program was developed on the basis of the focus group held, whereas a few leaders of the Kayoi-no-ba were invited. The experience of co-development of the program through the focus group may render the leaders more proactive in their approach to the participants. As such, this process may have undermined the generalizability of the program.

## 5. Conclusions

The study confirmed the high retention rate and the high percentage of exercise performed, whereas the low level of burden felt by the leader indicated that the study is feasible. A trend was observed toward improvement in health indicators, which may help prevent the progression of frailty. However, the effectiveness of the intervention remains unknown due to the lack of detection power and the comparison of one arm. In the future, on the basis of the correction of the points of improvement obtained in the current study, conducting a cluster RCT with power to confirm the effectiveness is necessary.

## Figures and Tables

**Table 1:** Overview of the intervention program.

	Performed by Leader	Performed by Participants
Content	Distribution of NCGG-HEPOP booklet. Distribution of exercise menu for 1 session for 10 min. Check the participant’s exercise record sheet on the day of the visit. Check once a week about the status of the participant’s exercise.	Read the NCGG-HEPOP booklet. Choose an exercise program based on the flow chart. Perform 10 min of daily exercise at home Describe goals, daily exercise records, and special notes in exercise record sheet Submit the exercise record sheet to the leader on the day of the Kayoi-no-ba meeting.

**Table 2:** Description of exercise content for each package of the NCGG-HEPOP.

Package	Exercise Type	Number of Sets	Number of Repetitions/ Implementation Time	Load
Strengthening package	Stretching the hamstrings	1	30 s each	-
	Stretching tight calf muscles	1	20 s each	-
	Squats	1	30 times	Body weight
	Standing training in a tandem position	1	30 s each	-
	Standing on one leg	1	30 s each	Body weight
	Marching in place	2	1 min	Body weight
Balance improvement package	Stretching the upper back and chest	3	10 s	-
	Stretching the arms and back	3	10 s	-
	Knee straightening exercise	2	10 times each	Body weight
	Standing heel raises	1	30 times	Body weight
	Standing up from a chair	1	30 times	Body weight
Inactivity prevention package	Stretching the quadriceps and front of the hip	1	30 s each	-
	Full body stretch	2	30 s	-
	Hip abduction exercise	3	20 times each	Body weight
	Twist exercise	3	10 times each	Body weight
	Drawing circles with the feet	2	10 times each	Body weight
	Standing heel raises	2	20 times	Body weight

Which package to implement was selected based on the flowchart. It was recommended that the relevant package be implemented once a day.

**Table 3:** Characteristics of the participants at baseline.

	All (*n* = 63)		Robust (*n* = 25)		Pre-Frail and Frail (*n* = 38)	
Age (mean/SD)	79.5	5.3	78.2	4.1	80.3	5.7
Body mass index (*n*/%)						
<18.5	5	7.9	2	8.0	3	7.9
18.5–24.9	51	81.0	21	84.0	30	78.9
≥25.0	7	11.1	2	8.0	5	13.2
Disease status (multiple answers) (*n*/%)						
Stroke	2	3.2	1	4.0	1	2.6
Cardiovascular disease	11	17.5	6	25.0	5	13.2
Diabetes mellitus	5	7.9	3	12.5	2	5.3
Respiratory disease	7	11.1	2	8.3	5	13.2
Musculoskeletal disorders	6	9.5	1	4.2	5	11.9
Number of medications (*n*/%)						
None	8	12.7	2	8.0	6	15.8
One or two	21	33.3	12	48.0	9	23.7
Three or four	25	39.7	6	24.0	19	50.0
Five or more	8	12.7	4	16.0	4	10.5

SD: standard deviation.

**Table 4:** Feasibility outcomes during the intervention and at the end of the intervention.

		All (*n* = 63)		Robust (*n* = 25)		Pre-Frail and Frail (*n* = 38)	
Date during the intervention period							
Percentage of leaders confirming implementation (mean/SD)		74.6	21.7	79.3	16.1	71.5	24.4
Percentage of exercise performed (mean/SD)		86.5	20.0	91.1	14.2	83.5	22.7
Data at the end of the intervention							
Satisfaction (*n*/%)	Extremely satisfied	21	33.3	11	45.8	10	26.3
	Satisfied	16	25.4	5	20.8	11	28.9
	Neutral	22	34.9	8	33.3	14	36.8
	Unsatisfied	2	3.2	0	0.0	2	5.3
	Extremely unsatisfied	0	0.0	0	0.0	0	0.0
	Missing	2	3.2	1	4.2	1	2.6

SD: standard deviation.

**Table 5:** Changes in health indicators before and after the intervention.

		All (*n* = 63)						Robust (*n* = 25)						Pre-Frail and Frail (*n* = 38)					
		*n*	Pre		Post		*p*	*n*	Pre		Post		*p*	*n*	Pre		Post		*p*
Frail	*n*/%	61	18	29.5	14	23.0	0.289	24	0	0.0	0	0.0	-	37	18	48.6	14	37.8	0.289
Risk assessment scale	Mean/SD	57	21.2	7.0	20.7	6.8	0.248	24	17.4	5.4	17.9	4.7	0.357	33	23.9	6.7	22.7	7.5	0.048
EQ5D5L	Median (25–75)	61	0.895	0.823–1.000	0.895	0.780–0.895	0.099	23	0.895	0.895–1.000	0.895	0.871–0.895	0.029	38	0.831	0.759–0.895	0.837	0.729–0.895	0.399
Grip strength (kg)	Mean/SD	59	22.3	4.1	22.8	4.4	0.106	25	21.5	4.7	22.4	4.5	0.091	34	22.9	3.5	23.1	4.4	0.669
Five times sit-to-stand test (seconds)	Median (25–75)	38	9.9	8.4–11.7	8.2	7.3–9.6	<0.001	19	9.1	7.7–10.8	7.8	7.0–9.5	0.001	19	11.4	9.2–13.6	9.1	7.9–10.1	0.001
MVPA (min/day)	Median (25–75)	51	31.7	15.4–49.9	28.3	15.4–42.7	0.484	22	37.7	26.6–52.5	33.6	22.9–60.4	0.758	29	23.6	8.1–44.6	20.4	14.4–38.5	0.452
Change in frequency of exercise (*n*/%)		63																	
	Increased				30	47.6					14	56.0					16	42.1	
	Slight/no change				31	49.2					10	40.0					21	55.3	
	Decreased				0	0.0					0	0.0					0	0.0	
	Missing				2	3.2					1	4.0					1	2.6	

MVPA; moderate to vigorous physical activity. Post-evaluation of MVPA was measured at 8 (±1) weeks after the start of the intervention. Changes in frequency of exercise are assessed at the end of the intervention and do not represent pre-intervention results.

## Data Availability

An anonymous analyzed data will be available to researchers upon reasonable request to the corresponding author.
